# Bio-enzymes for inhibition and elimination of *Escherichia coli* O157:H7 biofilm and their synergistic effect with sodium hypochlorite

**DOI:** 10.1038/s41598-019-46363-w

**Published:** 2019-07-09

**Authors:** Eun Seob Lim, Ok Kyung Koo, Min-Jeong Kim, Joo-Sung Kim

**Affiliations:** 1 Department of Food Biotechnology, Korea University of Science and Technology, 217, Gajeong-ro, Yuseong-gu, Daejeon 34113 Republic of Korea; 2 Department of Food and Nutrition, Gyeongsang National University, 501 Jinju-daero, Jinju, Gyeongsangnam-do 52828 Republic of Korea; 3 Institute of Agriculture and Life Science, Gyeongsang National University, 501 Jinju-daero, Jinju, Gyeongsangnam-do 52828 Republic of Korea; 4 Research Group of Consumer Safety, Research Division of Strategic Food Technology, Korea Food Research Institute, 245, Nongsaengmyeong-ro, Iseo-myeon, Wanju-gun, Jeollabuk-do 55365 Republic of Korea

**Keywords:** Biofilms, Biofilms, Pathogens, Pathogens

## Abstract

*Escherichia coli* O157:H7 is one of the most important pathogens worldwide. In this study, three different kinds of enzymes, DNase I, proteinase K and cellulase were evaluated for inhibitory or degrading activity against *E. coli* O157:H7 biofilm by targeting extracellular DNA, proteins, and cellulose, respectively. The cell number of biofilms formed under proteinase K resulted in a 2.43 log CFU/cm^2^ reduction with an additional synergistic 3.72 log CFU/cm^2^ reduction after NaClO post-treatment, while no significant reduction occurred with NaClO treatment alone. It suggests that protein degradation could be a good way to control the biofilm effectively. In preformed biofilms, all enzymes showed a significant reduction of 16.4–36.7% in biofilm matrix in 10-fold diluted media (*p* < 0.05). The sequential treatment with proteinase K, cellulase, and NaClO showed a significantly higher synergistic inactivation of 2.83 log CFU/cm^2^ compared to 1.58 log CFU/cm^2^ in the sequence of cellulase, proteinase K, and NaClO (*p* < 0.05). It suggests that the sequence of multiple enzymes can make a significant difference in the susceptibility of biofilms to NaClO. This study indicates that the combination of extracellular polymeric substance-degrading enzymes with NaClO could be useful for the efficient control of *E. coli* O157:H7 biofilms.

## Introduction

*Escherichia coli* O157:H7 is one of the most important foodborne pathogens worldwide, causing gastroenteritis, hemolytic uremic syndrome (HUS), hemorrhagic colitis and thrombotic thrombocytopenic purpura in susceptible groups such as children and elderly people^1^. It is generally highly associated with cattle, and contaminated fresh produce has been recently implicated in foodborne illness by *E. coli* O157:H7^2^. In addition, such foodborne outbreaks can also occur after consumption of food cross-contaminated with pathogens residing in food-associated environments, including production, transport, and cooking processes^3–5^.

Bacterial cells adhere to abiotic surfaces and produce film-like structures that protect the cells from environmental stresses, such as disinfection in a food processing environment^6–11^. This structure, called biofilm, can lead to serious problems during food production, distribution and consumption by cross-contamination^6,12–16^. Previous studies showed that biofilms of *E. coli* O157:H7 that form on food contact surfaces such as stainless steel were resistant to the drying environment^17^ and disinfection^18^.

In biofilms, bacterial cells produce extracellular polymeric substances (EPS) with extracellular DNA, protein and polysaccharides and form a slimy film surrounding the bacterial cells^19,20^. Additionally, EPS is involved in the attachment of cells to surfaces and in the formation of three-dimensional structures of biofilms and serves as a protective barrier to commercial disinfectants^21–24^. Therefore, for effective microbial control, a novel strategy for efficient inactivation of bacterial cells in biofilms is required.

Recently, methods using EPS-degrading enzymes have been attempted to disintegrate the EPS of biofilms^25^. Sadekuzzaman *et al*. (2015) proposed the use of deoxyribonuclease I (DNase I), lysostaphin (LS), α-amylase, lyase and lactonase for biofilm control^16^. Lequette *et al*. (2010) applied serine protease, papain, α-amylase, cellulase, and β-glucanase to control biofilms of isolates from various industrial origins and confirmed various susceptibility of the isolates to the enzymes^26^. Also, Kim *et al*. (2013) applied proteinase K, trypsin, subtilisin and dispersin B to biofilms formed on a fouled reverse osmosis membrane, and showed different efficiencies of these enzymes^25^.

Cellulose and curli fiber are the main constituents of EPS in *E. coli* biofilms^27–29^ and could be potential targets for effective biofilm control by enzymes. However, insufficient studies have been conducted regarding the effectiveness or effective treatment methods of enzymes in removing EPS of *E. coli* O157:H7 biofilm. Despite insufficient studies, Martins *et al*. (2012) showed that treatment with antimicrobial agents after enzyme treatment can effectively inactivate microbial cells in biofilms^30^. Such a method of using enzymes or combined with antimicrobial agents has yet to be studied for efficient inactivation of *E. coli* O157:H7 cells in biofilms.

Therefore, in this study, three enzymes, DNase I, proteinase K, and cellulase, targeting extracellular DNA, proteins, and cellulose, respectively, were evaluated for their ability to inhibit biofilm formation or degrade preformed biofilms of *E. coli* O157:H7 under different nutrient concentrations and the synergistic effect combined with NaClO, a major commercial disinfectant.

## Results

### Effect of enzymes on biofilm formation or biofilm developed on polystyrene microtiter plates

**Figure 1:**
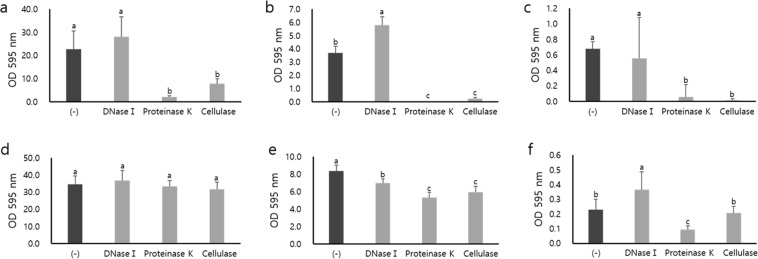
Quantification of *E. coli* O157:H7 biofilm matrix on polystyrene 96-well microtiter plates by Crystal Violet assay. Biofilms were formed in the presence of enzymes at 25 °C for 24 h (**a**~**c**), or preformed biofilms were treated with enzymes at 37 °C for 1 h (**d**~**f**) in undiluted (**a**,**d**), 10-fold diluted (**b**,**e**) and 50-fold diluted (**c**,**f**) BHI. The vertical lines represent the standard deviation of three independent experiments performed in triplicate. The different lowercase letters indicate significant differences at *p* < 0.05 using Tukey’s HSD.

To inhibit the biofilm formation of *E. coli* O157:H7, three enzymes, DNase I, proteinase K and cellulase, were added to the inoculum or to the preformed biofilms on the polystyrene microtiter plates in brain heart infusion broth (BHI) at different concentrations (none, 10-fold, and 50-fold diluted) (Fig. 1). When the inoculum was incubated in the presence of DNase I, no reduction in biofilm formation occurred compared to the absence of DNase I, regardless of the nutrient concentrations. However, proteinase K and cellulase treatment showed significantly reduced biofilm formation by 91.1–99.5% and 65.5–98.5%, respectively, regardless of BHI concentration (*p* < 0.05) (Fig. 1a–c). When the preformed biofilms were treated, DNase I, proteinase K, and cellulase treatment showed significant reductions of 16.4%, 36.7%, and 29.3%, respectively, in 10-fold diluted BHI (*p* < 0.05), while no significant reduction occurred for all the tested enzymes in undiluted medium (*p* > 0.05) (Fig. 1d,e). In addition, proteinase K showed a significant reduction of 60.9% in 50-fold diluted BHI (*p* < 0.05) (Fig. 1f). Overall, proteinase K was most effective for the inhibition of biofilm formation or the degradation of preformed biofilms, followed by cellulase and DNase I. The lower nutrient concentration resulted in a lower biofilm formation ability and a higher susceptibility to post-treated enzymes in general (Fig. 1).

To investigate the effect of proteinase K on the growth of *E. coli* O157:H7, it was incubated in BHI at 25 °C in the presence of proteinase K and the growth of planktonic cells was studied (Supplementary Fig. S1). There was no significant growth defect of cells in the presence of proteinase K (*p* > 0.05) (Supplementary Fig. S1).

### Combined treatment using enzymes followed by NaClO for the inhibition of biofilm development or removal of biofilm developed on stainless steel

**Figure 2:**
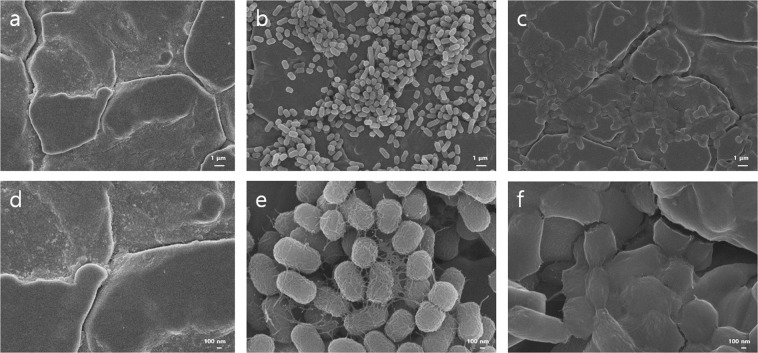
SEM images of *E. coli* O157:H7 biofilms on stainless steel surfaces: No inoculation (**a**,**d**); *E. coli* O157:H7 biofilm untreated (**b**,**e**) or treated with NaClO (**c**,**f**) at 20 ppm for 10 min. The biofilms were formed on a stainless steel surface in BHI at 25 °C for 24 h following 2 h preincubation for attachment. Magnifications and bar markers are ×10,000 and 1 μm long (**a**~**c**) or ×50,000 and 100 nm long (**d**~**f**), respectively.

**Figure 3:**
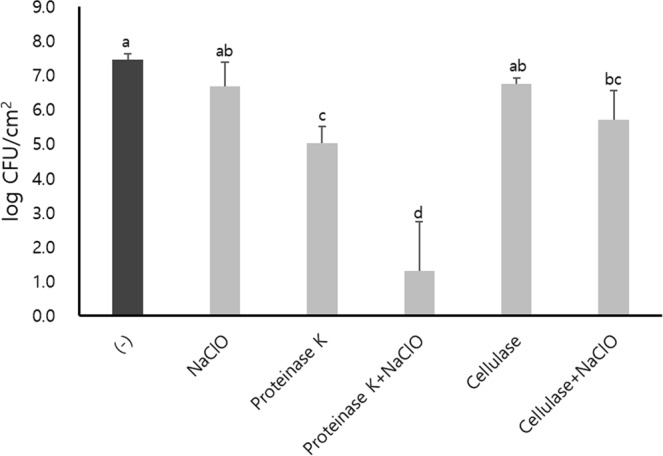
Numbers of *E. coli* O157:H7 viable cells on stainless steel surfaces grown in the presence of proteinase K or cellulase in BHI at 25 °C for 24 h and synergistic inactivation with NaClO post-treatment at 20 ppm for 10 min. The vertical lines represent the standard deviation of three independent experiments performed in duplicate. The different lowercase letters indicate significant differences at *p* < 0.05 using Tukey’s HSD.

**Figure 4:**
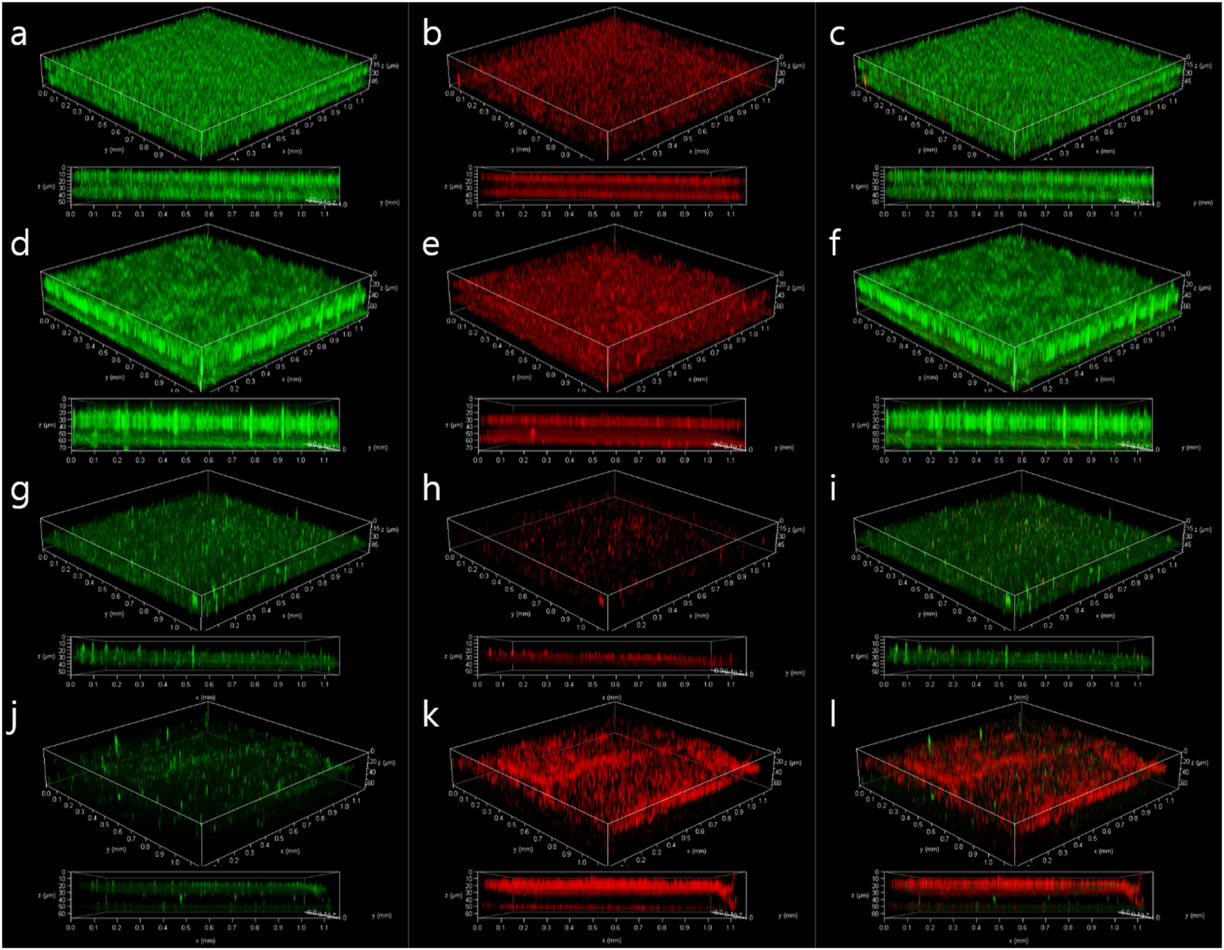
CLSM 3D and Z-stack images (upper and lower side of each set, respectively) of live (**a**,**d**,**g**,**j**), dead (**b**,**e**,**h**,**k**), and combined live and dead cells (**c**,**f**,**i**,**l**) of *E. coli* O157:H7 biofilms developed on stainless steel surfaces in BHI at 25 °C for 24 h. The samples were untreated (**a**~**c**), treated with NaClO at 20 ppm for 10 min after incubation (**d**~**f**), incubated in the presence of proteinase K (**g**~**i**) and incubated in the presence of proteinase K followed by NaClO treatment after incubation (**j**~**l**). Live (green) and dead (red) cells were stained with a LIVE/DEAD™ BacLight™ Bacterial Viability kit. The stainless steel surfaces are positioned underneath the biofilms in the images.

*E. coli* O157:H7 was incubated in the presence of stainless steel coupons submerged in BHI containing proteinase K or cellulase, then the biofilm formed on the coupon was exposed to sodium hypochlorite (NaClO), and the inactivation effects were studied with microscopic methods and viable counts. SEM analysis revealed that NaClO treatment alone caused a significant deformation of many cells such as flattening on stainless steel coupons (Fig. 2). However, NaClO treatment alone did not significantly affect the biofilm based on the viable counts (*p* > 0.05) (Fig. 3) and confocal laser scanning microscopy (CLSM) images (Fig. 4).

Proteinase K treatment showed a significant reduction (*p* < 0.05) of 2.43 log CFU/cm^2^ and a great synergistic inactivation of 6.15 log CFU/cm^2^ with NaClO post-treatment (Fig. 3). In contrast, no reduction in biofilm cells was observed under cellulase treatment alone (*p* > 0.05), and the biofilm cells treated with cellulase followed by NaClO were significantly inactivated (*p* < 0.05), but by only 1.74 log CFU/cm^2^ (Fig. 3). Consistent with the viable counts, proteinase K treatment showed a significant decrease in the density of viable cells, and the density of dead cells was significantly increased in combination with NaClO post-treatment in the CLSM analysis (Fig. 4). To understand if such an inactivation effect was due to any corrosion by NaClO on the stainless steel surface, surface corrosion experiment was conducted (Supplementary Fig. S2). No significant changes were observed on the stainless steel surface before and after NaClO exposure (Supplementary Fig. S2).

**Figure 5:**
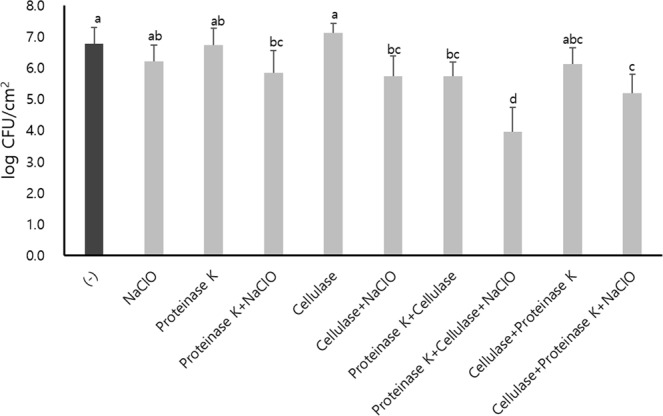
Numbers of *E. coli* O157:H7 viable cells in preformed biofilms after proteinase K, cellulase, or sequential treatment of both at 37 °C for 1 h each, or followed by NaClO treatment at 20 ppm for 10 min. The biofilms were formed on stainless steel surface in 10-fold diluted BHI at 25 °C for 24 h. The vertical lines represent the standard deviation of three independent experiments performed in duplicate. The different lowercase letters (**a**–**d**) indicate significant differences at *p* < 0.05 using Tukey’s HSD.

**Figure 6:**
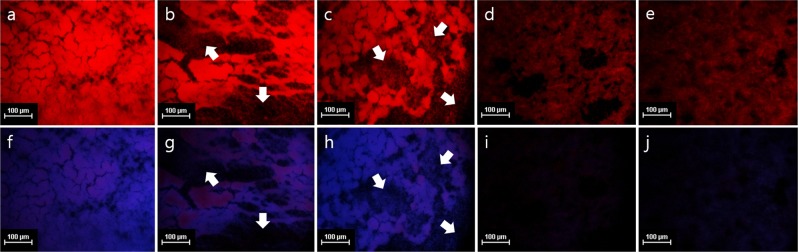
Fluorescence microscopy images of curli amyloid fibers with cellulose (a~e) and cellulose (f~j) of *E. coli* O157:H7 preformed biofilm matrix untreated (**a**,**f**) or treated with proteinase K (**b**,**g**), cellulase (**c**,**h**), proteinase K followed by cellulase (**d**,**i**) and cellulase followed by proteinase K (**e**,**j**) at 37 °C for 1 h for each enzyme after biofilm formation in 10-fold diluted BHI at 25 °C for 24 h. Curli amyloid fibers were stained by Congo Red (red) and cellulose was stained by Congo Red (red) and Calcofluor (blue). Arrows indicate the noticeable reduction in biofilm biomass. Bar markers are 100 μm long.

When enzymes were added to the preformed biofilm on stainless steel coupons in 10-fold diluted BHI, none of the proteinase K, cellulase, or NaClO treatment alone significantly reduced the number of viable cells (*p* > 0.05) (Fig. 5). However, the combined treatment using proteinase K followed by NaClO, cellulase followed by NaClO or proteinase K followed by cellulase showed a significant (*p* < 0.05) but limited inactivation with a maximum reduction of 1.05 log CFU/cm^2^. However, the sequential treatment of both enzymes followed by NaClO showed a notable reduction of viable cells. In particular, there was a considerable synergistic inactivation of 2.83 log CFU/cm^2^ in the order of proteinase K, cellulase, and NaClO (Fig. 5). Interestingly, a different enzyme sequential treatment in the order of cellulase, proteinase K, followed by NaClO was significantly less effective with only 1.58 log CFU/cm^2^ (*p* < 0.05). However, no significant difference was found between the two different treatments without NaClO (*p* > 0.05) (Fig. 5). Moreover, in the fluorescence microscopic analysis, such a sequential multiple enzyme treatment clearly showed a great reduction in the biofilm matrix compared to the single enzymes showing a limited reduction (Fig. 6).

Lastly, we confirmed the presence of genetic factors involved in biofilm formation (*csgD* and *flhDC*) and stress responses (*rpoS*, *oxyR*, *soxR*, *nemR*, and *rclR*) by PCR amplification of this strain (Supplementary Fig. S3).

## Discussion

Recently, methods using EPS-degrading enzymes have been studied for potential applications in biofilm control^25^. In this study, we tested the applicability or efficacy of those enzymes, DNase I, proteinase K, and cellulase for the prevention of biofilm formation or disruption of pre-existing biofilms of *E. coli* O157:H7.

DNase I was generally not effective in controlling *E. coli* O157:H7 biofilms in this study. Extracellular DNA is known to play an important structural role as a component of various bacterial biofilms and to protect bacterial cells from environmental stresses^20,31–37^. Some studies revealed that extracellular DNA produced by *E. coli* binds to the DNABII protein and increases the stability of biofilm^38,39^. Tetz and Tetz (2010) reported that in the presence of DNase I, the biofilm formation and antibiotic resistance of *E. coli* were reduced^40^. Furthermore, Nijland *et al*. (2010) have shown that the DNase (NucB) synthesized by *Bacillus licheniformis* dispersed biofilms of *E. coli*^41^. The lack of DNase-induced biofilm reduction in this study may represent the difference in EPS structures depending on the strains, causing a difference in susceptibility to DNase.

Previous studies have already demonstrated that the biofilms of many pathogens, such as *E. coli* O157:H7^42^, *Salmonella*^43^, *Listeria monocytogenes*^44^ and *Staphylococcus aureus*^45,46^, can be degraded by proteinase K. An extracellular protein fiber that constitutes the biofilm matrix of *E. coli* called curli is known to be one of the major components of *E. coli* biofilms and helps attach cells to abiotic surfaces and form biofilms^29,47–50^. Vacheva *et al*. (2012) have shown that protein/peptide factors involved in forming a biofilm are reduced by proteinase K^51^. CsgA, a major component of curli is also degradable by proteinase K although its fiber form is only partly degradable^52^. Therefore, in this study, proteinase K may have affected the function or assembly of curli and interfered with the initial attachment of inoculated cells, greatly preventing the biofilm formation of *E. coli* O157:H7 in our study (Fig. 3). The growth rate of *E. coli* O157:H7 in the presence of proteinase K was not significantly different (Supplementary Fig. S1), indicating that proteinase K did not affect the viability or growth rate of *E. coli* O157:H7 ATCC43894 and that the reduced biofilm formation with proteinase K treatment was not due to any growth defects. Our results strongly suggest that the proteinase K-mediated degradation of proteins such as curli can be an effective way to prevent biofilm formation or reduce the preformed biofilms of *E. coli* O157:H7.

Cellulose is known to play a role in surface attachment and biofilm construction^53^ and to protect biofilms against disinfectants in some bacteria^54,55^. Some studies showed that the biofilms of *Salmonella*^43^, *Pseudomonas aeruginosa*^56^, *P. flavescens*^57^*, P. fluorescens*^26^ and *Burkholderia cepacia*^58^ can be controlled by cellulase. Cellulose is also an important architectural element in *E. coli* biofilms^29,59–61^, which can be inhibited or degraded by cellulase^56–58^. Among different stages of biofilm formation, bacterial cellulose fibers are involved in irreversible attachment, which is a step that leads to the early development stage of biofilm formation and affects the maturation of biofilm^62,63^. Therefore, in our study, the cellulase present in the inoculum seems to have affected the formation of biofilm by interfering with the irreversible attachment stage. Additionally, the result of cellulase post-treatment strongly suggests that cellulose is the major architectural element of the mature biofilm of *E. coli* O157:H7 used in this study. However, proteinase K, a protease, seems to be more effective in the control of *E. coli* O157:H7 biofilm than cellulase belonging to polysaccharidase based on our study. This tendency is similar to the previous study of Lequette *et al*.^26^. Taken together, it seems that proteases are more efficient and cover a wider range of strains than polysaccharidases.

Some studies have shown that the biofilm forming ability of *E. coil* was reduced in minimal medium, similar to the results in our study^64^. Additionally, there have been reports that the addition of protein components in the growth medium increased polysaccharides in biofilms of *Proteus mirabilis*^65^, and excess nitrogen and carbon sources were used to synthesize additional extracellular proteins and polysaccharides in *Bacillus* spp.^66^. Therefore, we hypothesize that the lack of available nutrients may have resulted in the poor biosynthesis of biofilm-related factors such as extracellular proteins and polysaccharides. In addition, our study suggests that nutrient availability may affect the biofilm structure or integrity of *E. coli* O157:H7 based on the increased vulnerability of biofilms to enzyme post-treatment under nutrient-deficient conditions (Fig. 1).

NaClO, known as a disinfectant commonly used in a variety of food-associated environments^8,67^, has a broad disinfection range for various bacteria by inactivating enzymes necessary for the life cycle and by damaging cell membranes and DNA^68^. Our study demonstrates that NaClO treatment alone is limited in removing *E. coli* O157:H7 biofilm. Similarly, several studies have also shown that biofilm-forming microorganisms are resistant to disinfectants^10,11,69–74^. Corcoran *et al*. (2014) reported that the biofilm formed by *Salmonella* cannot be removed by NaClO^75^. Ryu *et al*. (2005) described that the EPS components of *E. coli* O157:H7 biofilm might serve as a protective barrier to NaClO^24^. Additionally, the efficacy of disinfection by NaClO was reduced by organic matter such as protein or cellulose^54,55,76^. From these studies, it is considered that the high resistance of biofilm cells against NaClO in this study may be due to the barrier effect of the EPS matrix and the reduction of disinfection efficacy by organic matter in the EPS matrix. Therefore, the degradation of EPS would be a good strategy to improve the efficacy of NaClO. From our results, it was confirmed that the biofilm of *E. coli* O157:H7 formed in the presence of proteinase K was greatly inactivated by subsequent NaClO treatment compared to the biofilm in the absence of proteinase K (Figs 3 and 4). This outcome suggests that proteinase K treatment combined with disinfectants such as NaClO can synergistically improve biofilm prevention or disinfection. Similarly, Cui *et al*. (2016) showed that *E. coli* O157:H7 biofilm was greatly reduced by thyme oil in the presence of proteinase K^42^. Because proteinase K degrades the various protein/peptide factors associated with biofilm formation^51^ and curli is one of the protein factors associated with early attachment and has protective properties in *E. coli* biofilm^48,50,77^, it is likely that proteins including curli are degraded by proteinase K. Ryu and Beuchat (2005) reported that *E. coli* O157:H7 became more resistant to chlorine in an environment that produces curli well^24^. Bap-mediated *Staphylococcus aureus* biofilm was dispersed by proteinase K^45^. Therefore, proteinase K may cause defects in the biofilm structure and reduce the barrier properties, thereby facilitating NaClO penetration and reducing the survivability of cells. Therefore, our data suggest that proteins may be good targets to remove to allow the efficient penetration of disinfectants such as NaClO to inactivate *E. coli* O157:H7 cells in biofilm. In addition, the increased sensitivity of biofilm cells to NaClO after exposure to proteinase K compared to cellulase strongly suggests that a proper choice of enzymes is important for efficient inactivation of biofilm cells using disinfectants (Fig. 3).

The biofilm CLSM images show a weak intensity at the middle of the biofilm (Fig. 4). Considering the dyes used in this study, the amount of DNA in cells or extracellular DNA could be much reduced in the weak intensity regions. A distinct phenotype depending on the region of the biofilm was also previously observed in non-pathogenic *E. coli*^60^.

Our study suggests that the sequential treatment using multiple enzymes can be more effective than single enzymes in removing a preformed biofilm (Fig. 5). Such an improved efficacy is also evident in the fluorescence microscopic analysis (Fig. 6). Furthermore, the differential inactivation efficacy depending on the treatment order of multiple enzymes prior to NaClO treatment strongly suggests that it is important for efficient inactivation of *E. coli* O157:H7 biofilm cells (Fig. 5). It may also reflect the structural or spatial distribution of biofilm constituents. Our data may suggest that proteins exist more commonly than cellulose in the outermost layer of biofilm matrix protecting cells. From the above results, it can be concluded that the appropriate combination and treatment order can increase the versatility and efficiency of enzymes in biofilm control.

In fact, the method of microbial control using enzymes is disadvantageous in terms of cost and stability for commercial use. In addition, enzyme activity is highly dependent on environmental factors such as temperature and is optimal only in limited conditions. When using protease, self-degradation causing instability should also be considered^78^. As a solution, enzyme activity can be stabilized by immobilizing enzymes on abiotic surfaces^78^. In addition, it will be cost-effective if sufficient stability is maintained after repetitive use through immobilization.

In conclusion, the biofilm formation of *E. coli* O157:H7 can be significantly inhibited in the presence of enzymes such as proteinase K or cellulase. In particular, biofilm inhibition can be synergistically enhanced by proteinase K combined with NaClO treatment. Additionally, sequential treatment using multiple enzymes followed by disinfectant can synergistically inactivate the cells in preformed biofilms. Accordingly, the combination of EPS-degrading enzymes with conventional disinfectants could be used as an alternative strategy for efficient control of biofilms produced by foodborne pathogens such as *E. coli* O157:H7 in the food-associated environment.

## Methods

### Bacterial strain and growth conditions

*E. coli* O157:H7 ATCC43894 (American Type Culture Collection, Manassas, VA, USA) was used in this study. The bacterial strain was inoculated in brain heart infusion broth (BHI, Merck, NJ, USA) and incubated at 37 °C for 18–24 h in a shaking incubator. The inoculum was prepared by diluting the overnight culture in BHI to approximately 10^7^ CFU/ml at an OD_600_ of 0.02–0.03, and the number of cells was confirmed by plating on tryptic soy agar (TSA, Merck) and incubating for 24 h at 37 °C.

### Evaluation of enzymatic effects on biofilm on polystyrene surface

Biofilm formation was performed on a polystyrene surface in a 96-well cell culture plate (SPL, Pocheon, Korea) and the biofilm matrix was quantified by crystal violet (CV) assay as previously described^5^. To examine the ability of enzymes to inhibit biofilm formation, 200 μl of inoculum with enzymes was incubated on a 96-well plate (SPL) at 25 °C for 24 h. For degradation activity against preformed biofilm, 200 μl of inoculum, as prepared above, was incubated on a 96-well plate at 25 °C for 24 h. Then, the wells were washed once by dispensing and aspirating 400 μl of phosphate buffered saline (PBS, Dongin, Seoul, Korea), post-treated with the enzymes diluted in BHI (200 μl), and incubated at 37 °C for 1 h. Final concentrations of enzymes were as follows: 0.1% (v/v) DNase I (Thermo Scientific™, Waltham, MA, USA), 1% (v/v) proteinase K (QIAGEN, Hilden, Germany), and 20 mg/ml cellulase (Duchefa Biochemie, Haarlem, The Netherlands). After incubation, the medium containing the enzymes was removed by pipetting and the wells were washed once with PBS. Then, 200 μl of CV solution (bioWORLD, Ohio, USA) diluted to 1% in deionized water (DW) was added and incubated for 30 min at room temperature (RT). After washing three times with PBS, 200 μl of absolute ethanol (EtOH, JT Baker, MA, USA) was added and incubated for 15 min at RT for destaining. After 100 μl of the destaining solution was transferred to a new 96-well plate, the absorbance was measured at 595 nm using an Infinite® M200 PRO NanoQuant microplate reader (Tecan, Männedorf, Switzerland). The final OD values at 595 nm were calculated by subtracting the OD value of the negative control well (incubating BHI only) from the OD values of the samples. When the measurement values exceeded an OD_595_ of 2.0, the samples were appropriately diluted in EtOH to an OD_595_ between 0.5–2.0, and the dilution factors were multiplied. All experiments were performed independently three times in triplicate.

### Combined treatment for removal of biofilms on stainless steel

Food grade stainless steel coupons (#304, 2 cm × 2 cm × 0.2 cm) were used as the surface for biofilm formation. Coupons were washed with 2% RBS™ 35 Concentrate (Thermo Scientific™) with sonication and rinsed with DW followed by EtOH. Washed coupons were dried in a dry oven and autoclaved at 121 °C for 15 min. To examine the synergistic effect of enzymes with sodium hypochlorite (NaClO; Junsei, Tokyo, Japan) on biofilm formation, 4.5 ml of the inoculum was inoculated in each well of a sterile 6-well plate (SPL) with a sterile stainless steel coupon and incubated at 25 °C for 2 h for cell adhesion. After the coupons were washed once with sterile PBS, 4.5 ml of the mixtures of fresh BHI with 1% proteinase K or with 20 mg/ml cellulase were added to each well with the coupon and incubated at 25 °C for 24 h. Fresh BHI without inoculum was used as a negative sample. For sequential treatment of preformed biofilms with enzymes followed by NaClO, stainless steel coupons were inoculated and incubated at 25 °C for 2 h, washed once with PBS, and 4.5 ml of fresh BHI was added and incubated at 25 °C for 24 h. After washing three times with PBS, 4.5 ml of the mixtures of fresh BHI with 1% proteinase K or with 20 mg/ml cellulase were added individually or sequentially and incubated at 37 °C for 1 h for each treatment. For NaClO treatment, the coupons were washed with PBS and treated with 4.5 ml of NaClO at 20 ppm in DW at RT for 10 min. PBS was used as a negative control. Then, the coupons were briefly rinsed once with PBS and vortexed in 15 ml of PBS with sterile glass beads for 60 s at maximum speed. Each sample was serially diluted, plated onto sorbitol MacConkey Agar (Oxoid, Wesel, Germany), and incubated at 37 °C up to 24 h for enumeration of viable cells attached to coupons. All experiments were performed independently three times in duplicate.

### Growth phenotype

Proteinase K was added at 1% to the inoculum described above and the samples were incubated at 25 °C and taken at 2 h intervals. The number of cells was confirmed by plating on TSA and incubating the plates for 24 h at 37 °C. All experiments were performed independently two times in triplicate.

### Surface corrosion experiment

Stainless steel coupons described above were treated with NaClO at 20 ppm at RT for 10 min, washed once with PBS, and dried in desiccator at RT. Stainless steel surfaces before and after treatment with NaClO were imaged by Dino-Lite AM4113T (AnMo Electronics, Hsinchu, Taiwan).

### Scanning electron microscopy (SEM) imaging

SEM was performed as previously described^79^. The samples were fixed with Karnovsky’s glutaraldehyde (0.05 M sodium cacodylate buffer (EMS, Hatfield, PA, USA), 2% paraformaldehyde (EMS), and 2% glutaraldehyde (EMS)) at 4 °C for 2 h. After washing with 0.05 M sodium cacodylate buffer twice, the samples were incubated in 1% osmium tetroxide (EMS) with 0.05 M cacodylate buffer at 4 °C for 2 h. Then, the samples were washed in DW and dehydrated in increasing alcohol concentrations (30%, 50%, 70%, 80%, 90% and 100%). The samples were dried with hexamethyldisilazane (EMS) for 18–24 h in a biosafety cabinet. After coating with Pt, the samples were examined by a scanning electron microscope (Carl Zeiss, Jena, Germany).

### Confocal laser scanning microscopy (CLSM) imaging

The samples were stained with a LIVE/DEAD™ *Bac*Light™ Bacterial Viability kit (Invitrogen™, CA, USA) according to the manufacturer’s instructions. Microscopic imaging was performed at 10 × magnification on a Leica TCS SP8 X confocal laser scanning microscope (CLSM, Leica, Heidelberg, Germany) using green (ex 490 nm, em 550 nm) and red (ex 570 nm, em 650 nm) wavelengths with 1 μm intervals to the z-axis. Images were merged and reconstructed to 3D and Z-stack images using the Leica Application Suite X software (Leica).

### Fluorescence microscopy imaging

The samples were stained with Congo Red (Sigma-Aldrich, St. Louis, MO, USA) for curli amyloid fibers plus cellulose and Calcofluor (Sigma-Aldrich) for cellulose using a modified protocol^80,81^. Briefly, the samples were stained in fresh alkalinized alcoholic Congo Red with Calcofluor dye (2% (w/v) NaCl (Duchefa Biochemie), 80% (v/v) EtOH (JT Baker), 0.01% (w/v) NaOH (Daejung, Siheung, Korea), 0.2% (w/v) Congo Red (Sigma-Aldrich), 250 μg/ml Calcofluor (Sigma-Aldrich)), incubated at RT for 30 min in the dark and dehydrated twice in absolute alcohol (JT Baker) at RT for 1 min each. Microscopic imaging was performed on an Eclipse 80i upright fluorescence microscope (Nikon, Tokyo, Japan) using blue (ex 360 nm, em 460 nm) and red (ex 560 nm, em 630 nm) wavelengths.

### PCR analysis

The inoculum described above was centrifuged at 13,000 × *g* for 5 min and the pellet was used for genomic DNA extraction using PrepMan™ Ultra Sample Preparation Reagent (Thermo Scientific, Waltham, USA) and PCR inhibitor removed by OneStep™ PCR inhibitor Removal Kit (Zymo Research, Irvine, USA). Target genes and PCR primer sequences are listed in Supplementary Table S1. The PCR reaction was composed of extracted DNA, primer pairs for each target gene, PCR-grade water, and Takara Ex Taq version 2.0 (Takara, Kusatsu, Japan). The PCR cycle was as follows: initial denaturation at 95 °C for 10 min, then 30 cycles of 1) Denaturation at 95 °C for 15 s, 2) Annealing and extension at 60 °C for 30 s. PCR products were separated by electrophoresis on 0.8% agarose gel in TAE buffer (40 mM Tris-HCl, 40 mM acetate, 1.0 mM EDTA), stained with Staining STAR (DyneBio, Seongnam, Korea) and confirmed with Gel Doc^TM^ EZ Imager (Bio-Rad, Richmond, USA).

### Statistical analyses

Statistical significance was determined by Tukey’s Honest Significant Difference (HSD) test and Student’s t-test procedure of Minitab 17 (Minitab Inc., PA, USA). The level of statistical significance was *p* < 0.05.

## Supplementary information

Bio-enzymes for inhibition and elimination of Escherichia coli O157:H7 biofilm and their synergistic effect with sodium hypochlorite

## Data Availability

All data generated or analysed during this study are included in this published article and its Supplementary information files.
