# Supplementary material for: Bio-enzymes for inhibition and elimination of Escherichia coli O157:H7 biofilm and their synergistic effect with sodium hypochlorite
Source: Sci Rep. 2019 Jul 9;9:9920. doi: 10.1038/s41598-019-46363-w (PMC6616338; doi:10.1038/s41598-019-46363-w)
Supplement: Supplementary file 1 — Bio-enzymes for inhibition and elimination of Escherichia coli O157:H7 biofilm and their synergistic effect with sodium hypochlorite [file 41598_2019_46363_MOESM1_ESM.pdf]

Bio-enzymes for inhibition and elimination of *Escherichia coli* O157:H7 biofilm and their synergistic effect with sodium hypochlorite

Eun Seob Lim[1,4], Ok Kyung Koo[2,3], Min-Jeong Kim[4], Joo-Sung Kim*[1,4]

[1]Department of Food Biotechnology, Korea University of Science and Technology, 217, Gajeong-ro, Yuseong-gu, Daejeon, 34113, Republic of Korea

[2]Department of Food and Nutrition, Gyeongsang National University, 501 Jinju-daero, Jinju, Gyeongsangnam-do, 52828, Republic of Korea

[3]Institute of Agriculture and Life Science, Gyeongsang National University, 501 Jinju-daero, Jinju, Gyeongsangnam-do, 52828, Republic of Korea

[4]Research Group of Consumer Safety, Research Division of Strategic Food Technology, Korea Food Research Institute, 245, Nongsaengmyeong-ro, Iseo-myeon, Wanju-gun, Jeollabuk-do, 55365, Republic of Korea

*Corresponding author

Joo-Sung Kim

Korea Food Research Institute

245, Nongsaengmyeong-ro, Iseo-myeon, Wanju-Gun,

Jeollabuk-do, Republic of Korea

Phone) +82-63-219-9266

Fax) +82-63-219-9876

Email) jskim@kfri.re.kr

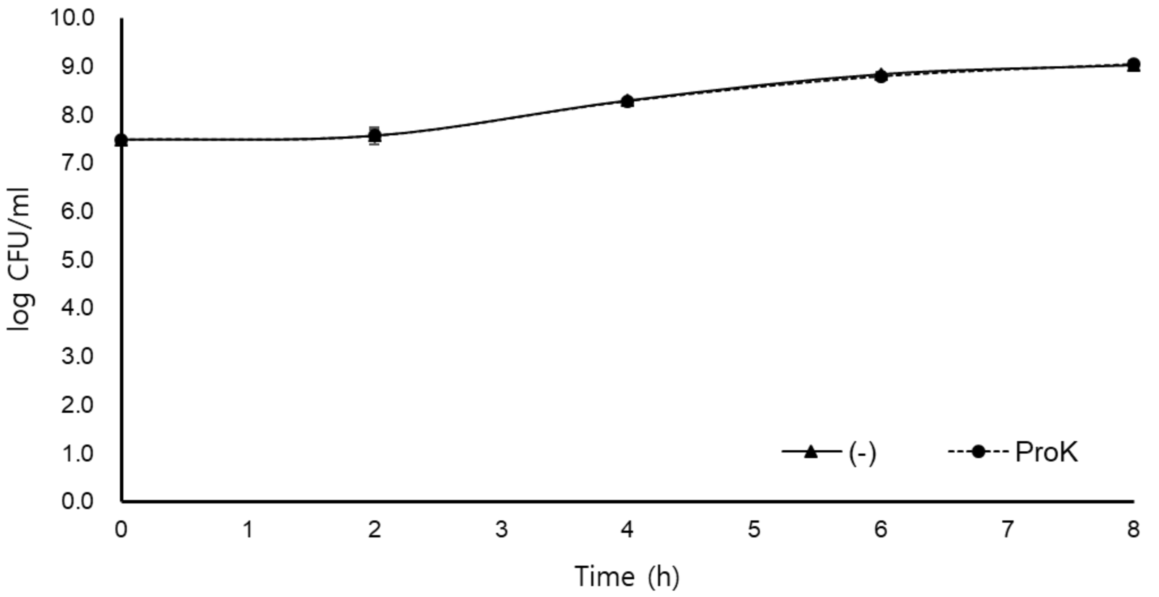

Figure S1. Growth patterns of *E. coli* O157:H7 ATCC43894 incubated with or without proteinase K at 25°C in 10 ml BHI.

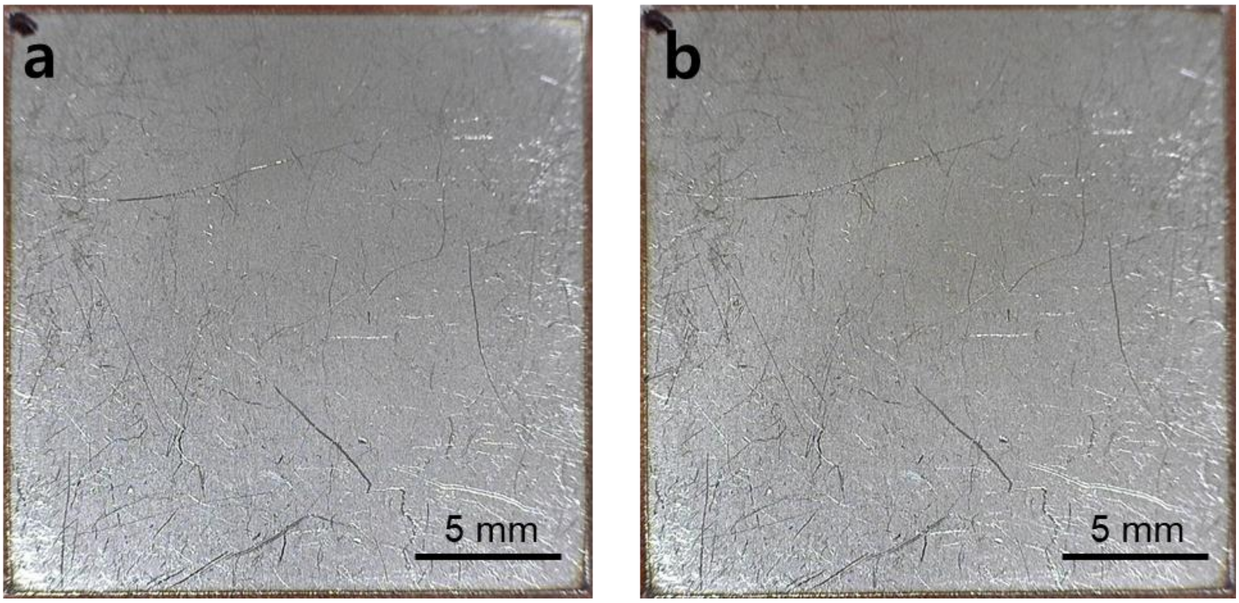

Figure S2. Images of stainless steel surface before (a) and after (b) treatment with sodium hypochlorite at 20 ppm for 10 min.

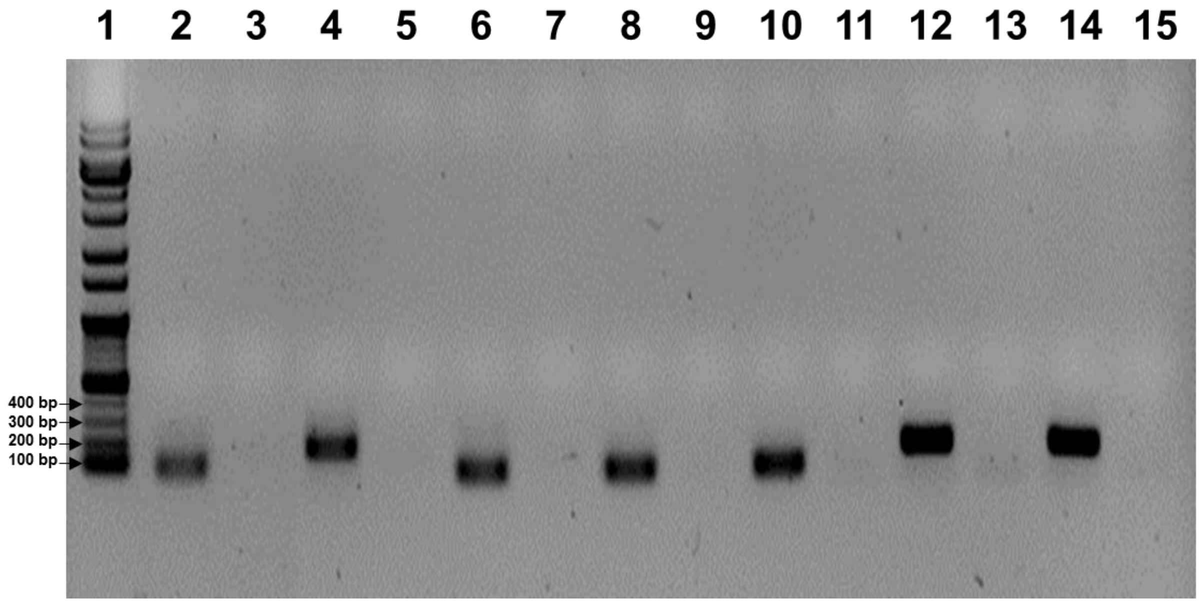

Figure S3. PCR amplification of biofilm formation and stress response related genes in *E. coli* O157:H7 ATCC43894. Lane 1: DNA marker; Lane 2: *csgD*; Lane 4: *flhDC*; Lane 6: *rpoS*; Lane 8: *oxyR*; Lane 10: *soxR*; Lane 12: *nemR*; Lane 14: *rclR*; Lanes 3, 5, 7, 9, 11, 13 and 15 indicate negative control of each gene.

Table S1. Primer set used in this study

Gene name	Primer sequence	Product length (bp)	Reference	Function (KEGG)
**Curli & Cellulose**				
csgD	F: 5'-CCGCTTGTGTCCGGTTTT-3'<br>R: 5'-GAGATCGCTCGTTCGTTGTTC-3'	97	[1]	Putative 2-component transcriptional regulator for 2nd curli operon
**Flagella**				
flhDC	F: 5'-GTGTAAAGACCCATTTCTATTTGTAAGGAC-3'<br>R: 5'-TGTGTTTCAGCAACTCGGAGGTATG-3'	202	[2]	Regulator of flagellar biosynthesis acting on class 2 operons; transcription initiation factor
**Stress response**				
rpoS	F: 5'-GCAGAGCATCGTCAAATGGCTGTT-3'<br>R: 5'-ATCTTCCAGTGTTGCCGCTTCGTA-3'	103	[3]	RNA polymerase, sigma S (sigma38) factor; synthesis of many growth phase related proteins
**ROS stress**				
oxyR	F: 5'-GAAGCACAGACCCACCAGTT-3'<br>R: 5'-CAAACAACGGCACTTCAATG-3'	109	[4]	Activator, hydrogen peroxide-inducible genes
soxR	F: 5'-GCATTAAAGCGCTGCTAACC-3'<br>R: 5'-ATTGCCGCTGTTACGGATAC-3'	116	[4]	Redox-sensing activator of *soxS*
**RCS stress**				
nemR	F: 5'-GGTCCTTCTATCACTACTTTCGCTCT-3'<br>R: 5'-GTTTTACTGTCAGGCAACCACTGAT-3'	204	[5]	Hypothetical protein
rclR	F: 5'-AAAAACATTCTCAGTTTGCTTCTGC-3'<br>R: 5'-CGCTATTTGTAGACGCAACTTTGTT-3'	201	[6]	Reactive chlorine species (RCS)-specific activator of the *rcl* genes

References

1. Chen, C. *et al.* Characteristics of *Escherichia coli* biofilm production, genetic typing, drug resistance pattern and gene expression under aminoglycoside pressures. *Environ. Toxicol. Pharmacol.* **30**, 5–10 (2010).

2. Wei, B. L. *et al.* Positive regulation of motility and *flhDC* expression by the RNA-binding protein CsrA of *Escherichia coli*. *Mol. Microbiol.* **40**, 245–256 (2001).

3. Ito, A., May, T., Kawata, K. & Okabe, S. Significance of *rpoS* during maturation of *Escherichia coli* biofilms. *Biotechnol. Bioeng.* **99**, 1462–1471 (2008).

4. Wang, S. *et al.* Transcriptomic response of *Escherichia coli* O157:H7 to oxidative stress. *Appl. Environ. Microbiol.* **75**, 6110–6123 (2009).

5. Gray, M. J., Wholey, W., Parker, B. W., Kim, M. & Jakob, U. NemR is a bleach-sensing transcription factor. *J. Biol. Chem.* **288**, 13789–13798 (2013).

6. Parker, B. W., Schwessinger, E. A., Jakob, U. & Gray, M. J. The RclR protein is a reactive chlorine-specific transcription factor in *Escherichia coli*. *J. Biol. Chem.* **288**, 32574–32584 (2013).
